# Maternal Mushroom Consumption During Pregnancy Is Associated With Decreased Risk of Peer Problems and Decreased Risk of Low Prosocial Behavior in 5‐Year‐Olds

**DOI:** 10.1111/jhn.70222

**Published:** 2026-02-26

**Authors:** Mai Quynh Nguyen, Yoshihiro Miyake, Keiko Tanaka, Shizuka Hasuo, Yoshitaka Nakamura, Hitomi Okubo

**Affiliations:** ^1^ R&D Division, Meiji Co. Ltd. Hachioji Tokyo Japan; ^2^ Wellness Science Labs, Meiji Holdings Co. Ltd. Hachioji Tokyo Japan; ^3^ Department of Epidemiology and Public Health Ehime University Graduate School of Medicine Toon Ehime Japan; ^4^ Integrated Medical and Agricultural School of Public Health Ehime University Toon Ehime Japan; ^5^ Research Promotion Unit, Translational Research Center Ehime University Hospital Toon Ehime Japan; ^6^ Center for Data Science Ehime University Matsuyama Ehime Japan; ^7^ Department of Healthcare Data Science Ehime University Graduate School of Medicine Toon Ehime Japan; ^8^ Department of Nutritional Epidemiology and Behavioural Nutrition Graduate School of Medicine The University of Tokyo Bunkyo Tokyo Japan

**Keywords:** behavioral problems, Japanese children, maternal diet, mushrooms, pregnancy, vegetables

## Abstract

**Background:**

Edible mushrooms have long been recognized for their nutritional value. In the Kyushu Okinawa Maternal and Child Health Study, a prebirth cohort study, we examined the association between maternal mushroom intake during pregnancy and risk of childhood behavioral problems in Japanese 5‐year‐olds.

**Methods:**

A total of 1199 mother–child pairs were included as study subjects. In the baseline survey, dietary intake was assessed using a diet history questionnaire. In the follow‐up survey, emotional problems, conduct problems, hyperactivity problems, peer problems, and low prosocial behavior were assessed using the parent‐reported version of the Strengths and Difficulties Questionnaire. Adjustments were made for a priori*–*selected non‐dietary confounders and potentially related dietary factors.

**Results:**

A significant inverse exposure–response association was observed between maternal mushroom consumption during pregnancy and risk of childhood peer problems (adjusted odds ratio [OR] between extreme quartiles, 0.58; 95% confidence interval [CI]: 0.31–1.07, *p* for trend = 0.02) and low prosocial behavior (adjusted OR between extreme quartiles, 0.64; 95% CI: 0.43–0.95, *p* for trend = 0.02).

**Conclusion:**

Higher maternal intake levels of mushrooms during pregnancy may be associated with a decreased risk of peer problems and a decreased risk of low prosocial behavior in 5‐year‐old children.

## Summary

- Maternal diet during pregnancy is associated with child development.
- Mushroom consumption was associated with a decreased risk of peer problems and a decreased risk of low prosocial behavior in children.
- Intake of mushrooms might be preventive against child behavioral problems development.

**Abbreviations**
CI: confidence interval
DHQ: diet history questionnaire
IQR: interquartile range
KOMCHS: Kyushu Okinawa Maternal and Child Health Study
OR: odds ratio
SDQ: Strengths and Difficulties Questionnaire

## 1 Introduction

Various edible mushrooms, such as shiitake (*Letinula edodes*) and enoki (*Flammulina velutipes*), have long been recognized for their nutritional values [1, 2, 3]. They are documented as a good source of protein (containing all of the essential amino acids), fiber, vitamins (B_1_, B_2_, B_12_, C, and D), and minerals (potassium, phosphorus, and iron) while being low in fat and cholesterol‐free [2, 3]. The presence of bioactive substances like polysaccharides (e.g., ß‐glucans), unsaturated fatty acids, phenolic compounds, and nucleic acid derivatives contributes to the documented health‐related properties of mushrooms [1, 2]. Previously, utilizing the data from the Kyushu Okinawa Maternal and Child Health Study (KOMCHS), in which the parent version of the Strengths and Difficulties Questionnaire (SDQ) was used for child behavior assessment, we showed that higher maternal intake levels of vitamin B_2_ [4] and calcium [5] during pregnancy were associated with a reduced risk of emotional problems in the born children. Maternal intake of monounsaturated fatty acids, α‐linolenic acid, and linoleic acid was positively related to childhood emotional problems [6]. We have also observed inverse relationships between maternal vitamin C intake during pregnancy and childhood conduct problems [7], between maternal intake of vitamin B_6_ [4], vitamin C [7], calcium [5], and magnesium [8] during pregnancy and childhood hyperactivity problems, as well as between maternal intake of folate, vitamin B_6_ [4], and vitamin C [7] during pregnancy and childhood low prosocial behavior. To the best of our knowledge, no epidemiological investigation has examined the association between mushroom intake during pregnancy and risk of childhood behavioral problems. In this study, we addressed this issue by investigating the association between maternal intake of mushrooms during pregnancy and risk of behavioral problems in 5‐year‐old children using data from the KOMCHS.

## 2 Materials and Methods

### 2.1 Study Population

The KOMCHS is a prospective prebirth cohort study that was designed to clarify the risk and preventive factors for maternal and child health problems, such as allergy, developmental disorders, and depressive symptoms. The details of the baseline KOMCHS survey were previously described [9]. In the baseline survey, eligible subjects were women in one of the seven prefectures on Kyushu Island in southern Japan, which has a total population of approximately 13.26 million, or in Okinawa Prefecture, which has a population of nearly 1.37 million, who were pregnant between April 2007 and March 2008. We asked 423 obstetric hospitals in the eight prefectures to provide information leaflets explaining the KOMCHS, an application form to participate in the KOMCHS, and a self‐addressed stamped return envelope to as many pregnant women as possible. Pregnant women who intended to participate in the KOMCHS mailed the application form to the data management center. Using the contact information in this form, research technicians gave each pregnant woman a detailed explanation of the KOMCHS by telephone and sent a self‐administered questionnaire after obtaining verbal consent. Ultimately, 1757 pregnant women between 5 and 39 weeks of gestation provided written informed consent to participate in the KOMCHS and answered a self‐administered questionnaire in the baseline survey. There were multiple waves of surveys conducted in the KOMCHS. Among the 1757 pregnant women from baseline survey, 1590, 1527, 1430, 1362, 1305, 1264, and 1201 mother–child pairs participated in the second (post‐delivery), third (approximately 4 months postpartum), fourth (approximately 12 months postpartum), fifth (approximately 24 months postpartum), sixth (approximately 36 months postpartum), seventh (approximately 48 months postpartum), and eighth (approximately 60 months postpartum) surveys, respectively. Two pairs were excluded due to the lack of data on household income. The final analyses comprised 1199 mother–child pairs. The KOMCHS was approved by the ethics committees at the Faculty of Medicine, Fukuoka University (committee's reference number, 319) and the Ehime University Graduate School of Medicine (committee's reference number, 1504012).

### 2.2 Measurements

For each survey, study participants completed self‐administered questionnaires and mailed them to the data management center. The research technicians then completed missing data or clarified illogical data through telephone interviews. The first part of the questionnaire at baseline elicited information on maternal age, gestation, region of residence, number of children, maternal and paternal educational levels, household income, and depressive symptoms. Depressive symptoms were assessed using a Japanese version of the Center for Epidemiologic Studies Depression Scale (CES‐D). The total CES‐D score ranged from 0 to 60, and depressive symptoms were defined as being present when a subject had a CES‐D score ≥ 16 [10]. The second part of the questionnaire at baseline was a semi‐quantitative, comprehensive diet history questionnaire (DHQ) to assess dietary habits during the preceding month [11, 12, 13, 14, 15, 16, 17]. Estimates of daily food intake (among 150 foods), energy, and selected nutrients were calculated using an ad hoc computer algorithm for the DHQ based on the Standard Tables of Food Composition in Japan [18]. Energy‐adjusted intake calculated using the residual method was used for the analyses [19]. The questionnaire in the second survey gathered data regarding the infant's sex, birth weight, date of birth, and maternal smoking status during pregnancy. Information on household smoking and breastfeeding duration was obtained from the questionnaire until the fourth survey. In the eighth survey, children's behavioral problems were assessed using a Japanese parent‐reported version of the SDQ, which was designed to assess the behavior and emotions of 3‐ to 16‐year‐old children [20]. The well‐validated SDQ [20, 21, 22] consists of five scales: an emotional problems scale, a conduct problems scale, a hyperactivity problems scale, a peer problems scale, and a prosocial scale. These scales were scored using five items each, resulting in 25 items in total. In the peer problems scale the following items were included: “Rather solitary, tends to play alone,” “Has at least one good friend,” “Generally liked by other children,” “Picked on or bullied by other children,” and “Gets on better with adults than with other children.” For the prosocial scale the items were: “Considerate of other people's feelings,” “Shares readily with other children (treats, toys, pencils, etc.),” “Helpful if someone is hurt, upset or feeling ill,” “Kind to younger children,” and “Often volunteers to help others (parents, teachers, and other children).” Each item was rated using the following 3‐point scale: “not true” (0); “somewhat true” (1); and “certainly true” (2). Positively worded items were reverse‐scored. The items on each scale were added together to generate a score from 0 to 10. A high score on the prosocial scale reflects strengths, whereas high scores on the other 4 scales indicate difficulties. These scale scores were then categorized as normal, borderline, or abnormal based on cut‐off points that had previously been reported in a sample of Japanese children [23].

### 2.3 Statistical Analyses

The 5 scale scores were dichotomized, comparing children with borderline and abnormal scores to children with normal scores. We defined emotional problems, conduct problems, hyperactivity problems, peer problems, or low prosocial behavior as present when a child had a borderline or abnormal score in the respective scale (> 3, > 3, > 5, > 3, and < 6, respectively). Intake of each of the dietary factors under study was categorized at quartile points based on its distribution among the 1199 mothers. A priori, we selected maternal age, gestation at baseline, region of residence at baseline, number of children at baseline, maternal and paternal education, household income, maternal depressive symptoms during pregnancy, maternal alcohol intake during pregnancy, maternal smoking during pregnancy, child's birth weight, child's sex, secondhand smoke exposure at home during the first year of life, and breastfeeding duration as potential non‐dietary confounding factors. We used maternal age, gestation, and birth weight as continuous variables. The potential dietary confounding factors that were significantly associated with any childhood behavioral problems in the present population were additionally adjusted. Namely, citrus fruit and cow's milk were adjusted for in analyses of the associations with emotional problems [7, 24]; citrus fruit was adjusted for in analyses of the associations with conduct problems [7]; vegetables other than green and yellow vegetables, total fruits [7], and total soy products [25] were adjusted for in analyses of the associations with hyperactivity problems; total soy products [25] was adjusted for in analyses of the associations with peer problems; and total vegetables was adjusted for in analyses of the associations with low prosocial behavior [7]. Sample size was determined by the available cohort. Given the observational design, adequacy of the sample size for multivariable logistic regression was assessed using the events‐per‐variable (EPV) approach [26]. Models included 15–17 covariates, with outcome events ranging from 103 to 350, resulting in EPV values of 9.1, 14.6, 8.7, 6.4, and 21.9 for emotional, conduct, hyperactivity, peer problems, and prosocial behavior, respectively. While a minimum EPV of 10 has been traditionally recommended, evidence suggests that models with slightly lower EPV remain valid when covariates are pre‐specified and overfitting is minimized [27]. In addition, post hoc considerations indicate that with a total sample of 1199 participants, the study had adequate power (≥ 80%; two‐sided *α* = 0.05) to detect small‐to‐moderate associations, depending on outcome prevalence. Specifically, the minimum detectable odds ratios (ORs) were approximately 1.8 for outcomes with low prevalence (~10%) and approximately 1.3–1.4 for more prevalent outcomes (~30%–40%). Overall, the sample size was considered adequate to detect meaningful differences in the outcomes examined and to support the planned analyses. Multivariable logistic regression analysis was conducted to estimate the adjusted ORs and 95% confidence intervals (CIs) for each behavioral problem according to the intake quartiles for mushrooms, and the lowest quartile was used as the reference. The trend of association was assessed using a logistic regression model assigning consecutive integers (1–4) to the intake quartiles for mushrooms, and two‐sided *p* values less than 0.05 were considered statistically significant. All computations were performed using the SAS software package version 9.4 (SAS Institute Inc., Cary, NC, USA).

## 3 Results

Among the 1199 children who were 59–71 months age, the prevalence values of emotional problems, conduct problems, hyperactivity problems, peer problems, and low prosocial behavior were 12.9%, 19.4%, 13.1%, 8.6%, and 29.2%, respectively. The median maternal age and gestation at baseline were 32.0 (interquartile range [IQR], 29.0–34.0) years and 17.0 (IQR, 14.0–21.0) weeks, respectively. Median maternal daily consumption of total energy during pregnancy was 7127 (IQR, 6117–8465) kJ, and median daily energy‐adjusted intake levels of mushrooms were 8.2 (IQR, 4.6–15.9) g. Maternal mushroom consumption during pregnancy was inversely associated with maternal depressive symptoms during pregnancy, maternal daily total energy consumption, maternal smoking during pregnancy, and smoking in the household during the first year of life of the born child. Mothers who had higher level of mushroom consumption were more likely to live in Kyushu areas other than Fukuoka prefecture, have a higher number of educational years, and breastfed their children for a longer period of time (Table 1).

**TABLE 1:** Characteristics of 1199 mother–child pairs, according to quartile (Q) of maternal mushroom intake during pregnancy.

	Total (*n* = 1199)^a^	Mushroom^b^				
		Q1	Q2	Q3	Q4	*p* for trend^c^
Baseline characteristics						
Maternal age, years	32.0 (29.0–34.0)	32.0	31.0	32.0	32.0	0.09
Gestation, weeks	17.0 (14.0–21.0)	17.0	17.0	17.0	18.0	0.59
Region of residence, %						0.005
Fukuoka Prefecture	57.8	61.9	56.0	61.0	52.3	
Other than Fukuoka Prefecture in Kyushu	32.8	25.8	32.3	33.0	40.0	
Okinawa Prefecture	9.4	12.4	11.7	6.0	7.7	
Number of living children born to the same mother, %						0.41
0	40.4	41.5	45.7	36.0	38.3	
1	40.0	35.8	37.7	46.3	40.3	
≥ 2	19.6	22.7	16.7	17.7	21.3	
Maternal education, years, %						0.004
< 13	20.9	25.8	21.7	19.3	16.7	
13–14	33.3	35.5	31.0	31.0	35.7	
≥ 15	45.9	38.8	47.3	49.7	47.7	
Paternal education, years, %						0.05
< 13	28.6	33.4	28.0	26.0	27.0	
13–14	14.4	14.4	13.3	17.3	12.7	
≥ 15	57.0	52.2	58.7	56.7	60.3	
Household income, yen/year, %						0.12
< 4,000,000	32.2	35.1	34.7	28.0	31.0	
4,000,000–5,999,999	37.5	38.5	34.7	37.0	39.7	
≥ 6,000,000	30.4	26.4	30.7	35.0	29.3	
Maternal depressive symptoms during pregnancy, %	18.2	20.7	21.0	16.0	15.0	0.03
Maternal alcohol intake during pregnancy, %	13.2	13.0	12.3	13.3	14.0	0.66
Maternal daily intake						
Total energy, kJ	7127 (6117–8465)	7816	6427	7201	7210	0.04
Mushrooms, g^d^	8.2 (4.6–15.9)					
Total vegetables, g^d^	187.2 (139.0–264.4)					
Vegetables other than green and yellow vegetables, g^d^	111.2 (79.3–158.9)					
Total fruits, g^d^	125.3 (80.8–185.8)					
Citrus fruit, g^d^	19.7 (7.0–46.2)					
Total soy products, g^d^	46.9 (30.0–70.2)					
Cow's milk, g^d^	78.3 (31.4–147.7)					
Characteristics at the postnatal assessment						
Maternal smoking during pregnancy, %	7.3	12.0	7.3	5.0	4.7	0.0003
Birth weight, g	3012 (2772–3246)	2990	3020	3033	3024	0.11
Male sex, %	47.4	44.2	50.7	48.0	46.7	0.71
Breastfeeding duration, months, %						< 0.0001
< 6	10.8	16.4	10.7	10.3	6.0	
≥ 6	89.2	83.6	89.3	89.7	94.0	
Smoking in the household during the first year of life, %	27.4	34.5	25.3	24.7	25.3	0.02

^a^ Values are medians (interquartile ranges) for continuous variables and percentages of subjects for categorical variables.

^b^ The intake range of each quartile was as follows: Q1 (< 4.6 g), Q2 (4.6– < 8.2 g), Q3 (8.2– < 15.9 g), and Q4 (≥ 15.9 g).

^c^ For continuous variables, a linear trend test was used; for categorical variables, a Mantel–Haenszel *χ* ^2^‐test was used.

^d^ Food intake level was adjusted for total energy intake using the residual method.

Following the adjustment for potential non‐dietary and dietary confounding factors, a significant trend of inverse exposure–response association was observed between maternal mushroom intake during pregnancy and risk of childhood peer problems (the adjusted OR between extreme quartiles was 0.58; 95% CI: 0.31–1.07, *p* for trend = 0.02; Table 2). Compared with maternal consumption of mushrooms during pregnancy in the first quartile, consumption in the fourth quartile was independently related to a reduced risk of low prosocial behavior in children and the inverse linear trend was statistically significant (adjusted OR between extreme quartiles, 0.64; 95% CI: 0.43–0.95; *p* for trend = 0.02). There were no measurable associations between maternal consumption of mushrooms during pregnancy and childhood emotional, conduct, or hyperactivity problems.

**TABLE 2:** Odds ratios (ORs) and 95% confidence intervals (CIs) for behavioral problems assessed by the Strengths and Difficulties Questionnaire in 1199 5‐year‐old children by quartiles of maternal mushroom intake during pregnancy.

Variables^a^	Emotional problems		Conduct problems		Hyperactivity problems		Peer problems		Low prosocial behavior	
	Risk (%)	Adjusted OR (95% CI)^b^	Risk (%)	Adjusted OR (95% CI)^c^	Risk (%)	Adjusted OR (95% CI)^d^	Risk (%)	Adjusted OR (95% CI)^e^	Risk (%)	Adjusted OR (95% CI)^f^
Mushroom intake										
Q1 (2.9)	15.1	1.00	20.4	1.00	15.4	1.00	11.0	1.00	32.8	1.00
Q2 (6.2)	12.0	0.80 (0.49–1.31)	26.0	1.34 (0.90–2.01)	15.3	0.91 (0.57–1.46)	11.0	0.98 (0.58–1.67)	33.3	0.94 (0.66–1.35)
Q3 (12.2)	11.7	0.91 (0.55–1.50)	14.3	0.65 (0.41–1.01)	11.0	0.72 (0.43–1.19)	6.0	0.55 (0.29–1.00)	27.7	0.78 (0.54–1.13)
Q4 (26.2)	13.0	0.95 (0.58–1.56)	17.0	0.87 (0.56–1.34)	10.7	0.72 (0.42–1.22)	6.3	0.58 (0.31–1.07)	23.0	0.64 (0.43–0.95)
*p* for trend		0.96		0.10		0.14		0.02		0.02

Abbreviations: CI, confidence interval; OR, odds ratio; Q, quartile.

^a^ Quartile medians in g/day adjusted for energy intake using the residual method are given in parentheses.

^b^ Adjustment for maternal age, gestation at baseline, region of residence at baseline, number of children at baseline, maternal and paternal education, household income, maternal depressive symptoms during pregnancy, maternal alcohol intake during pregnancy, maternal smoking during pregnancy, child's birth weight, child's sex, postnatal secondhand smoke exposure at home during the first year of life, breastfeeding duration, citrus fruit, and cow's milk consumption during pregnancy.

^c^ Adjustment for maternal age, gestation at baseline, region of residence at baseline, number of children at baseline, maternal and paternal education, household income, maternal depressive symptoms during pregnancy, maternal alcohol intake during pregnancy, maternal smoking during pregnancy, child's birth weight, child's sex, postnatal secondhand smoke exposure at home during the first year of life, breastfeeding duration, and citrus fruit consumption during pregnancy.

^d^ Adjustment for maternal age, gestation at baseline, region of residence at baseline, number of children at baseline, maternal and paternal education, household income, maternal depressive symptoms during pregnancy, maternal alcohol intake during pregnancy, maternal smoking during pregnancy, child's birth weight, child's sex, postnatal secondhand smoke exposure at home during the first year of life, breastfeeding duration, and consumption of vegetables other than green and yellow vegetables, total fruits, and total soy products during pregnancy.

^e^ Adjustment for maternal age, gestation at baseline, region of residence at baseline, number of children at baseline, maternal and paternal education, household income, maternal depressive symptoms during pregnancy, maternal alcohol intake during pregnancy, maternal smoking during pregnancy, child's birth weight, child's sex, postnatal secondhand smoke exposure at home during the first year of life, breastfeeding duration, and total soy product consumption during pregnancy.

^f^ Adjustment for maternal age, gestation at baseline, region of residence at baseline, number of children at baseline, maternal and paternal education, household income, maternal depressive symptoms during pregnancy, maternal alcohol intake during pregnancy, maternal smoking during pregnancy, child's birth weight, child's sex, postnatal secondhand smoke exposure at home during the first year of life, breastfeeding duration, and total vegetable consumption during pregnancy.

Maternal consumption of mushrooms during pregnancy was significantly correlated with maternal intake of vitamin C, folate, and vitamin B_6_ during pregnancy (Pearson's correlation coefficient: 0.23, *p* < 0.0001; 0.39, *p* < 0.0001; and 0.35, *p* < 0.0001, respectively), which were previously found to be associated with reduced risk of childhood low prosocial behavior [4, 7]. After additional adjustment for maternal intake of vitamin C or folate or vitamin B_6_ during pregnancy as continuous variables, the inverse association between maternal mushroom intake during pregnancy and childhood low prosocial behavior remained significant (adjusted ORs between extreme quartiles were 0.64 [95% CI: 0.43–0.96; *p* for trend = 0.02], 0.68 [95% CI: 0.46–1.01; *p* for trend = 0.04], and 0.67 [95% CI: 0.45–0.99; *p* for trend = 0.04], respectively).

## 4 Discussion

To the best of our knowledge, the current pre‐birth cohort study was the first to show independent inverse associations between higher maternal mushroom intake during pregnancy and risk of childhood peer problems and low prosocial behavior.

Although there are no previous studies on the relationship between mushroom consumption and child behavior, it has been reported that mushroom consumption has a protective effect against depressive symptoms in a cohort study of 87,822 Korean individuals [28].

The inverse association between maternal mushroom consumption during pregnancy and childhood low prosocial behavior persisted following the adjustment for maternal intake of vitamin C or folate or vitamin B_6_, suggesting that the observed association is unlikely to be ascribed to the presence of vitamin C or folate or vitamin B_6_ in the consumed mushrooms. Vitamin D, which is abundant in mushrooms [2], on the other hand, might be an important factor. Vitamin D affects brain development by acting on processes like cellular proliferation, differentiation, calcium signaling, neurotrophism, and neuroprotection [29, 30]. It is suggested that vitamin D may play a role in protecting children with social risk against antisocial behavior through its regulation of serotonin synthesis by acting on two different tryptophan hydroxylase (TPH) genes: *TPH1* and *TPH2* [31, 32]. According to a study of 300 children aged 11–12 years from Philadelphia, vitamin D moderates the association between early social adversity and multiple antisocial outcomes as higher social adversity was associated with greater antisocial behavior among vitamin D–insufficient, but not vitamin D–sufficient, children [33]. Another study utilizing mother–child data from the Environmental Influences on Child Health Outcomes (ECHO) cohort, in which child behavior was assessed using the SDQ or Child Behavior Checklist, and data were harmonized using a crosswalk conversion, shows evidence of an association between lower gestational vitamin D and childhood behavioral problems [34]. In particular, vitamin D concentrations in prenatal or cord blood were inversely associated with externalizing behavior T‐scores in middle childhood (6–13 years); and in a sensitivity analysis restricted to those with vitamin D assessed in prenatal maternal samples, vitamin D was inversely associated with externalizing and total behavioral problems in early childhood (1.5–5 years) [34]. It is, however, important to acknowledge that the observed results might be due to the contribution of nutrients other than vitamin D.

Methodological strengths of the present study include data collection from a relatively large sample over a long period of time extending from pre‐birth to 5 years old, and the adjustment for potential non‐dietary, as well as dietary confounding factors.

Certain limitations, however, should be kept in mind when interpreting the results of this study. Despite the adjustment for numerous potential confounding factors, the possibility of residual confounding cannot be entirely ignored. As for dietary assessment, the DHQ was designed to assess dietary intake for 1 month before the questionnaire was completed and could only give approximate consumption. In addition, the subjects in this study completed the DHQ anywhere between the fifth and 39th week of pregnancy. Therefore, any non‐differential exposure misclassification would have led to an attenuation of the measures of association. Here we reported the results of analysis considering the effects driven by only one dietary component—mushrooms—among the total of 150 foods that DHQ covers, which could have been obscured by the effects of other foods when considering them all as a whole—for instance in dietary pattern analysis. In regard to the SDQ, the parent‐reported data might have been biased. Further, although the dichotomization cut‐off points for the five outcomes under study were chosen based on a previous study conducted in Japan [23], it is uncertain whether they were reasonable. The possibility of non‐differential outcome misclassification might have altered the observed association toward the null.

Of the 1757 pregnant women who completed the baseline survey for this study between April 2007 and March 2008, 556 mother–child pairs were excluded because they did not participate in the eighth survey. We observed no evident differences between these 556 non‐participants and the 1201 participants in the eighth survey in terms of the distribution for number of children, depressive symptoms during pregnancy, or alcohol intake during pregnancy. Compared with non‐participants, the participants in the eighth survey were more likely to be older, have participated in the baseline survey earlier in gestation, live in Fukuoka Prefecture, report high maternal and paternal educational levels, and have a high household income. As the number of pregnant women who received the leaflet explaining the KOMCHS, an application form, and a set of self‐addressed and stamped return envelope at the 423 collaborating obstetric hospitals was unidentified, we were not able to calculate the participation rate at baseline. Among the 1757 participants at baseline, 978 lived in Fukuoka Prefecture. Data collected by the government of Fukuoka Prefecture revealed that there were 46,393 children born in 2007 and 46,695 children born in 2008, which suggests that the participation rate in the baseline survey of the KOMCHS must have been low. Therefore, the present study subjects were probably not representative of Japanese women in the general population. For instance, according to a population census conducted in 2000 in Fukuoka Prefecture, the proportions of women aged 30–34 years with < 13 years, 13–14 years, ≥ 15 years, and an unknown number of years of education were 52.0%, 31.5%, 11.8%, and 4.8%, respectively [35], while the corresponding figures for the present study were 20.9%, 33.3%, 45.9%, and 0.0%, respectively.

## 5 Conclusion

The present prebirth cohort study showed that a higher maternal intake level of mushrooms during pregnancy was independently associated with a decreased risk of peer problems and a decreased risk of low prosocial behavior in 5‐year‐old children. We acknowledge the need for additional epidemiological studies and studies investigating the mechanisms underlying the observed preventive association.

## Author Contributions

Conceptualization: Yoshihiro Miyake and Keiko Tanaka. Data curation: Mai Quynh Nguyen, Yoshihiro Miyake, Keiko Tanaka, Shizuka Hasuo, and Yoshitaka Nakamura. Formal analysis: Mai Quynh Nguyen, Yoshihiro Miyake, and Shizuka Hasuo. Funding acquisition: Yoshihiro Miyake and Keiko Tanaka. Investigation: Yoshihiro Miyake and Keiko Tanaka. Methodology: Yoshihiro Miyake, Keiko Tanaka, and Hitomi Okubo. Project administration: Yoshihiro Miyake. Resources: Yoshihiro Miyake and Hitomi Okubo. Software: Mai Quynh Nguyen, Yoshihiro Miyake, and Shizuka Hasuo. Validation: Mai Quynh Nguyen, Yoshihiro Miyake, Keiko Tanaka, and Shizuka Hasuo. Visualization: Mai Quynh Nguyen and Shizuka Hasuo. Writing – original draft: Mai Quynh Nguyen and Yoshihiro Miyake. Writing – review and editing: Mai Quynh Nguyen, Yoshihiro Miyake, and Keiko Tanaka.

## Ethics Statement

The KOMCHS was approved by the ethics committees at the Faculty of Medicine, Fukuoka University (committee's reference number, 319) and the Ehime University Graduate School of Medicine (committee's reference number, 1504012).

## Consent

All participants provided written informed consent.

## Conflicts of Interest

Mai Quynh Nguyen and Shizuka Hasuo were employed by Meiji Co. Ltd. and Meiji Holdings Co. Ltd. Yoshitaka Nakamura was employed by Meiji Co. Ltd. Keiko Tanaka and Yoshihiro Miyake were financially supported by Meiji Co. Ltd. and Meiji Holdings Co. Ltd. Hitomi Okubo has no conflicts of interest. Hitomi Okubo belongs to the Department of Nutritional Epidemiology and Behavioural Nutrition, Graduate School of Medicine, The University of Tokyo, which is a social cooperation program with Ajinomoto Co. Inc. However, Ajinomoto Co. Inc. was not involved in any part of this study.

## Data Availability

The datasets analyzed during the current study are not publicly available because the steering committee and the participants did not approve unrestricted data sharing.

## References

[1] W. M. Breene , “Nutritional and Medicinal Value of Specialty Mushrooms,” Journal of Food Protection 53, no. 10 (1990): 883–895. 31018285 10.4315/0362-028X-53.10.883

[2] A. Assemie and G. Abaya , “The Effect of Edible Mushroom on Health and Their Biochemistry,” International Journal of Microbiology 2022 (2022): 8744788. 35369040 10.1155/2022/8744788 PMC8967584

[3] C. Tang , P. C. X. Hoo , L. T. H. Tan , et al., “Golden Needle Mushroom: A Culinary Medicine With Evidenced‐Based Biological Activities and Health Promoting Properties,” Frontiers in Pharmacology 7 (2016): 474. 28003804 10.3389/fphar.2016.00474 PMC5141589

[4] Y. Miyake , K. Tanaka , H. Okubo , S. Sasaki , and M. Arakawa , “Maternal B Vitamin Intake During Pregnancy and Childhood Behavioral Problems in Japan: The Kyushu Okinawa Maternal and Child Health Study,” Nutritional Neuroscience 23, no. 9 (2020): 706–713. 30453854 10.1080/1028415X.2018.1548139

[5] K. Takahashi , K. Tanaka , Y. Nakamura , et al., “Calcium Intake During Pregnancy Is Associated With Decreased Risk of Emotional and Hyperactivity Problems in Five‐Year‐Old Japanese Children,” Nutritional Neuroscience 24, no. 10 (2021): 762–769. 31690246 10.1080/1028415X.2019.1676971

[6] Y. Miyake , K. Tanaka , H. Okubo , S. Sasaki , and M. Arakawa , “Maternal Fat Intake During Pregnancy and Wheeze and Eczema in Japanese Infants: The Kyushu Okinawa Maternal and Child Health Study,” Annals of Epidemiology 23, no. 11 (2013): 674–680. 24094480 10.1016/j.annepidem.2013.08.004

[7] Y. Miyake , K. Tanaka , H. Okubo , S. Sasaki , and M. Arakawa , “Maternal Consumption of Vegetables, Fruit, and Antioxidants During Pregnancy and Risk for Childhood Behavioral Problems,” Nutrition 69 (2020): 110572. 31563826 10.1016/j.nut.2019.110572

[8] Y. Miyake , K. Tanaka , H. Okubo , S. Sasaki , A. Tokinobu , and M. Arakawa , “Maternal Metal Intake During Pregnancy and Childhood Behavioral Problems in Japan: The Kyushu Okinawa Maternal and Child Health Study,” Nutritional Neuroscience 25, no. 8 (2022): 1641–1649. 33568010 10.1080/1028415X.2021.1885241

[9] Y. Miyake , K. Tanaka , H. Okubo , S. Sasaki , and M. Arakawa , “Fish and Fat Intake and Prevalence of Depressive Symptoms During Pregnancy in Japan: Baseline Data From the Kyushu Okinawa Maternal and Child Health Study,” Journal of Psychiatric Research 47, no. 5 (2013): 572–578. 23391129 10.1016/j.jpsychires.2013.01.012

[10] L. S. Radloff , “The CES‐D Scale: A Self‐Report Depression Scale for Research in the General Population,” Applied Psychological Measurement 1, no. 3 (1977): 385–401.

[11] S. Sasaki , F. Ushio , K. Amano , et al., “Serum Biomarker‐Based Validation of a Self‐Administered Diet History Questionnaire for Japanese Subjects,” Journal of Nutritional Science and Vitaminology 46, no. 6 (2000): 285–296. 11227800 10.3177/jnsv.46.285

[12] M. Shiraishi , M. Haruna , M. Matsuzaki , R. Murayama , and S. Sasaki , “Validity of a Diet History Questionnaire Estimating β‐Carotene, Vitamin C and α‐Tocopherol Intakes in Japanese Pregnant Women,” International Journal of Food Sciences and Nutrition 64, no. 6 (2013): 694–699. 23506338 10.3109/09637486.2013.775225

[13] M. Shiraishi , M. Haruna , M. Matsuzaki , R. Murayama , S. Kitanaka , and S. Sasaki , “Validity of a Self‐Administered Diet History Questionnaire for Estimating Vitamin D Intakes of Japanese Pregnant Women,” Maternal & Child Nutrition 11, no. 4 (2015): 525–536. 24118748 10.1111/mcn.12063 PMC6860190

[14] M. Shiraishi , M. Haruna , M. Matsuzaki , R. Murayama , Y. Yatsuki , and S. Sasaki , “Estimation of Eicosapentaenoic Acid and Docosahexaenoic Acid Intakes in Pregnant Japanese Women Without Nausea by Using a Self‐Administered Diet History Questionnaire,” Nutrition Research 33, no. 6 (2013): 473–478. 23746563 10.1016/j.nutres.2013.04.002

[15] M. Shiraishi , M. Haruna , M. Matsuzaki , R. Murayama , S. Sasaki , and S. Murashima , “Validity and Reproducibility of Folate and Vitamin B(12) Intakes Estimated From a Self‐Administered Diet History Questionnaire in Japanese Pregnant Women,” Nutrition Journal 11 (2012): 15. 22420377 10.1186/1475-2891-11-15 PMC3324379

[16] S. Kobayashi , S. Honda , K. Murakami , et al., “Both Comprehensive and Brief Self‐Administered Diet History Questionnaires Satisfactorily Rank Nutrient Intakes in Japanese Adults,” Journal of Epidemiology 22, no. 2 (2012): 151–159. 22343326 10.2188/jea.JE20110075 PMC3798594

[17] S. Sasaki , R. Yanagibori , and K. Amano , “Self‐Administered Diet History Questionnaire Developed for Health Education: A Relative Validation of the Test‐Version by Comparison With 3‐Day Diet Record in Women,” Journal of Epidemiology 8, no. 4 (1998): 203–215. 9816812 10.2188/jea.8.203

[18] Science and Technology Agency. Standard Tables of Food Composition in Japan, 5th ed.; Printing Bureau of the Ministry of Finance: Tokyo, Japan, 2005. (In Japanese).

[19] W. Willett and M. J. Stampfer , “Total Energy Intake: Implications for Epidemiologic Analyses,” American Journal of Epidemiology 124, no. 1 (1986): 17–27. 3521261 10.1093/oxfordjournals.aje.a114366

[20] R. Goodman , “The Strengths and Difficulties Questionnaire: A Research Note,” Journal of Child Psychology and Psychiatry 38, no. 5 (1997): 581–586. 9255702 10.1111/j.1469-7610.1997.tb01545.x

[21] R. Goodman , H. Meltzer , and V. Bailey , “The Strengths and Difficulties Questionnaire: A Pilot Study on the Validity of the Self‐Report Version,” European Child & Adolescent Psychiatry 7, no. 3 (1998): 125–130. 9826298 10.1007/s007870050057

[22] R. Goodman and S. Scott , “Comparing the Strengths and Difficulties Questionnaire and the Child Behavior Checklist: Is Small Beautiful?,” Journal of Abnormal Child Psychology 27, no. 1 (1999): 17–24. 10197403 10.1023/a:1022658222914

[23] T. Matsuishi , M. Nagano , Y. Araki , et al., “Scale Properties of the Japanese Version of the Strengths and Difficulties Questionnaire (SDQ): a Study of Infant and School Children in Community Samples,” Brain and Development 30, no. 6 (2008): 410–415. 18226867 10.1016/j.braindev.2007.12.003

[24] M. Q. Nguyen , Y. Miyake , K. Tanaka , et al., “Maternal Consumption of Dairy Products During Pregnancy Is Associated With Decreased Risk of Emotional Problems in 5‐Year‐Olds: The Kyushu Okinawa Maternal and Child Health Study,” Nutrients 14, no. 22 (2022): 4713. 36432404 10.3390/nu14224713 PMC9697969

[25] Y. Miyake , K. Tanaka , H. Okubo , S. Sasaki , A. Tokinobu , and M. Arakawa , “Maternal Consumption of Soy and Isoflavones During Pregnancy and Risk of Childhood Behavioural Problems: The Kyushu Okinawa Maternal and Child Health Study,” International Journal of Food Sciences and Nutrition 72, no. 8 (2021): 1118–1127. 33792472 10.1080/09637486.2021.1904844

[26] P. Peduzzi , J. Concato , E. Kemper , T. R. Holford , and A. R. Feinstein , “A Simulation Study of the Number of Events Per Variable in Logistic Regression Analysis,” Journal of Clinical Epidemiology 49, no. 12 (1996): 1373–1379. 8970487 10.1016/s0895-4356(96)00236-3

[27] E. Vittinghoff and C. E. McCulloch , “Relaxing the Rule of Ten Events Per Variable in Logistic and Cox Regression,” American Journal of Epidemiology 165, no. 6 (2007): 710–718. 17182981 10.1093/aje/kwk052

[28] S. K. Park , C. M. Oh , J. H. Ryoo , and J. Y. Jung , “The Protective Effect of Mushroom Consumption on Depressive Symptoms in Korean Population,” Scientific Reports 12, no. 1 (2022): 21914. 36536111 10.1038/s41598-022-26549-5 PMC9763420

[29] C. Di Somma , E. Scarano , L. Barrea , et al., “Vitamin D and Neurological Diseases: An Endocrine View,” International Journal of Molecular Sciences 18, no. 11 (2017): 2482. 29160835 10.3390/ijms18112482 PMC5713448

[30] D. W. Eyles , “Vitamin D: Brain and Behavior,” JBMR Plus 5, no. 1 (2021): e10419. 33553986 10.1002/jbm4.10419 PMC7839822

[31] C. Moul , C. Dobson‐Stone , J. Brennan , D. Hawes , and M. Dadds , “An Exploration of the Serotonin System in Antisocial Boys With High Levels of Callous‐Unemotional Traits,” PLoS One 8, no. 2 (2013): e56619. 23457595 10.1371/journal.pone.0056619 PMC3574002

[32] R. P. Patrick and B. N. Ames , “Vitamin D Hormone Regulates Serotonin Synthesis. Part 1: Relevance for Autism,” FASEB Journal 28, no. 6 (2014): 2398–2413. 24558199 10.1096/fj.13-246546

[33] O. Choy and A. Raine , “Vitamin D Sufficiency Attenuates the Effect of Early Social Adversity on Child Antisocial Behavior,” Psychological Medicine 52, no. 16 (2021): 4106–4115. 10.1017/S0033291721001069 33762031

[34] M. M. Melough , M. Li , G. Hamra , et al., “Greater Gestational Vitamin D Status Is Associated With Reduced Childhood Behavioral Problems in the Environmental Influences on Child Health Outcomes Program,” Journal of Nutrition 153, no. 5 (2023): 1502–1511. 37147034 10.1016/j.tjnut.2023.03.005 PMC10367223

[35] Statistics Bureau Ministry of Public Management Home Affairs Posts and Telecommunications. Labour Force Status of Population, Industry (Major Groups) of Employed Persons, and Education, Fukuoka‐Ken. In 2000 Population Census of Japan; Statistics Bureau, Ministry of Public Management, Home Affairs, Posts and Telecommunications: Tokyo, Japan, 2002; 3‐2‐40. (In Japanese).

